# Impact on postoperative complications of combined prehabilitation targeting co-existing smoking, malnutrition, obesity, alcohol drinking, and physical inactivity: a systematic review and meta-analysis of randomised trials

**DOI:** 10.12688/f1000research.150880.2

**Published:** 2025-04-08

**Authors:** Line N Lydom, Sofie Anne-Marie S Jensen, Susanne V Lauridsen, Mette Rasmussen, Robin Christensen, Ulla N Joensen, Jacob Rosenberg, Hanne Tønnesen

**Affiliations:** 1 The Parker Institute, Bispebjerg and Frederiksberg Hospital, Frederiksberg, Denmark; 2 Centre for Perioperative Optimization, Department of Surgery, Herlev Hospital, Herlev, Denmark; 3 Department of Health Sciences, Lunds Universitet, Lund, Skåne County, Sweden; 4 Cochrane Denmark & Centre for Evidence-Based Medicine Odense (CEBMO), Department of Clinical Research, University of Southern Denmark, Odense, Denmark; 5 Department of Clinical Medicine, University of Copenhagen, Copenhagen, Capital Region of Denmark, Denmark; 6 Department of Urology, Copenhagen University Hospital Rigshospitalet, Copenhagen, Denmark

**Keywords:** Prehabilitation, Postoperative complications, Surgery, Lifestyle

## Abstract

**Background:**

This study aimed to compare the effect on postoperative complications of combined prehabilitation targeting predefined co-existing risky SNAP factors with usual preoperative routines in surgical patients.

**Methods:**

This systematic review followed the PRISMA 2020 guideline and the protocol (CRD42022282611). Five databases were searched from inception to November 7, 2022 for randomised controlled trials on prehabilitation targeting ≥2 predefined risky lifestyles compared with usual preoperative routines. Risky lifestyles included Smoking, Nutrition (malnutrition and/or BMI>25), risky Alcohol intake, and Physical inactivity (SNAP). Primary outcome was postoperative complications ≤30 days. Cochrane’s risk-of-bias tool 2 was used and meta-analyses were conducted. GRADE was used to assess certainty of evidence.

**Results:**

The search resulted in 20,862 records. At full-text screening, only two (120 participants) of 24 identified trials on combined SNAP intervention had ≥2 predefined risk factors and were included. One (n=110) on intensive physical and brief nutritional intervention to frail patients with colorectal cancer resection reported complication rates of 45% in both groups (relative risk (RR) 1.00, 95% CI 0.66 to 1.51). The other study (n=10, subgroup) on intensive alcohol and smoking intervention in patients with bladder cancer undergoing radical cystectomy, reported complications in 3/7 vs 3/3 participants. The meta-analysis estimated a RR of 0.79 (95% CI 0.41 to 1.51, I ^2^ 51%).

**Conclusion:**

Two small of the 24 trials on prehabilitation targeted co-existing and predefined risky SNAP factors and the effect on postoperative complications is very uncertain. Future prehabilitation research involving patient needs is warranted.

## Introduction

Surgery is a pivotal part of the treatment of numerous medical conditions. The estimated global volume of surgical procedures was 313 million procedures in 2012 and this has since increased. ^1^ Development of complications is still a major challenge worldwide ^2^^,^^3^ despite the improvements in perioperative care in the last decades including improved surgical and anaesthesiologic techniques, preoperative interventions targeting co-morbidities, and enhanced postoperative recovery programmes. ^4^^–^^6^ The consequences of complications include prolonged recovery, reduced quality of life and life expectancy, as well as increased health care costs. ^7^^–^^10^

Postoperative morbidity is in part related to preoperative modifiable risk factors such as smoking, ^11^^,^^12^ nutrition (overweight/obesity, malnutrition), ^13^^–^^15^ risky alcohol drinking, ^16^ and physical inactivity ^17^^–^^19^ (SNAP). The SNAP factors compromise several organ functions of importance for successful outcome after surgery. ^13^^,^^20^^–^^23^ While daily smoking, risky alcohol intake, or malnutrition are followed by a general 50% increased complication rate across different types of surgery, ^11^^,^^13^^,^^16^ overweight or obesity are associated with increased complications to a minor degree. ^24^^,^^25^ Low physical activity level is associated with increased risk of complications, length of stay and postoperative mortality. ^18^^,^^26^^–^^29^

The SNAP factors are modifiable by prehabilitation, ^20^^,^^22^^,^^30^^,^^31^ but the impact on postoperative morbidity differs among the SNAP factors. ^32^^,^^33^ Only the most intensive programmes targeting smoking, alcohol, and malnutrition reduce complication rates by half. ^13^^,^^32^^,^^33^ Recent systematic reviews have shown an improvement after preoperative physical training programmes of functional capacity both pre- and postoperatively, however, no effect on postoperative complications has been shown. ^34^^,^^35^ The effect of overweight or obesity interventions have only poorly been investigated outside bariatric surgery. ^36^ Until now, the large majority of the evidence from SNAP interventions are based on investigating one risky SNAP factor intervention at a time. ^37^^,^^38^ This is despite of up to half of hospital patients have co-existing SNAP factors, ^39^^–^^41^ such as smoking and overweight or frailty involving both malnutrition and physical inactivity which significantly potentiate the risk at surgery. ^14^^,^^42^ For example Park et al. found that the combination of being obese and smoking at the same time potentiated the risk of complications after surgery. ^14^ As the risk of the two risk factors in combination seems to potentiate each other, it points even more towards the relevance of investigating the effect of combined prehabilitation.

This study aimed to compare the effect on postoperative complications of combined prehabilitation targeting predefined co-existing risky SNAP factors with usual preoperative routines in surgical patients. We hypothesised the combined prehabilitation targeting predefined co-existing risky SNAP factors would reduce the postoperative complications compared to usual preoperative routines.

## Methods

This systematic review and meta-analysis was conducted and reported according to the Cochrane Handbook for Systematic Reviews of Interventions ^43^ and in line with PRISMA (Preferred Reporting Items for Systematic Reviews and Meta-Analyses) ^44^ and AMSTAR (Assessing the methodological quality of systematic reviews) ^45^ Guidelines. The protocol was registered on PROSPERO ( CRD42022282611) before retrieval of data. 

### Eligibility criteria

We included randomised controlled trials enrolling populations of adult patients (≥18 years) undergoing any surgical intervention and having predefined co-existing risky SNAP factors, defined as at least two of the five risky SNAP factors (daily smoking, alcohol intake > 2 drinks daily (= above 24 g ethanol) or 14 drinks weekly, malnutrition (defined as either weight loss of 10-15% within the last 6 months, BMI < 18.5, Subjective Global Assessment (SGA) Grade C, Nutritional Risk Screening (NRS) > 5, or preoperative serum albumin < 30 g/l), BMI > 25 and physical activity < 4 hours/week), or as described by the authors and receiving a combined prehabilitation initiated preoperatively and targeting those lifestyles. Populations in need for combined frailty intervention were accepted if the frailty screening tool involved at least two of the predefined risky lifestyles of interest in this review. Studies were excluded if their population did not have predefined risky lifestyles. Only face-to-face interventions (physical or online) were included. Intensive interventions were defined as having at least four sessions, each lasting longer than ten minutes and including education, motivational and (if relevant) pharmaceutical support. ^32^^,^^46^ The intervention was compared with usual preoperative routines.

### Information sources & search strategy

We searched Medline, Embase, Web of Science, Cochrane Central Register of Controlled Trials, and CINAHL on March 3, 2021 with an update on November 7, 2022. Our search strategy for MEDLINE was developed in consultation with an information specialist from the Cochrane Anaesthesia Group. It was combined with the Cochrane Highly Sensitive Search Strategy, with use of the sensitivity- and precision-maximising version, for identifying RCTs ^47^ (see extended data file). Similar strategies were developed for the other databases. The search was performed without restrictions for language or publication year. In addition, we performed a simple keyword search (“ *multimodal prehabilitation surgery RCT*”) in Google Scholar with a check-up of the first 200 references and a snowball search by manually scanning the reference lists of included trials and of topic relevant systematic reviews.

### Study selection

Screening of articles was conducted using Covidence® with an institutional subscription. Two reviewers screened all titles and abstracts, independently. Likewise, all full text articles were screened by two other reviewers. Any disagreements were discussed and resolved within the author group.

### Data collection and items

Data extraction was performed in collaboration of two reviewers with all discrepancies discussed and resolved within the author group. The following data were extracted from all studies into a pre-defined Excel-sheet: authors, year of publication, country, number of patients, patient characteristics (age, sex, ASA-score, SNAP factors), type of surgery, indication for surgery, intervention characteristics (targeted risky SNAP factors, intensity of intervention, preoperative duration, compliance measured as meeting adherence, additional interventions), definition of control groups, drop-outs, and length of follow-up.

The primary outcome was complications within 30 postoperative days, defined by requiring treatment and categorised according to a standardised methodology e.g., the Clavien-Dindo classification, ^48^ comprehensive complication index, ^49^ or as described by the authors.

We only included published randomized controlled trials (RCTs) including cluster and pilot RCTs. Secondary outcomes were extracted if present in the studies: length of stay (LoS), readmissions, adverse events of the lifestyle intervention, successful risk reduction perioperatively and long-term, and effect on patient reported long-term (one year) quality of life (QoL).

Successful risk reduction was defined as quit smoking, quit drinking, no overweight or obesity, no malnutrition, physical activity >4h/week as well as change of at least 1 step on the ASA-score ^50^ regarding risky lifestyles or any change of at least 1 unhealthy lifestyle. For long-term successful risk reduction same definitions were applied except for alcohol which needed to be below risky limits instead of zero intake exclusively.

When doubts about extraction of data, trial authors were contacted by email for clarification. For studies that included a subset of eligible participants, we obtained data for only the subgroup of interest.

### Risk of bias assessment

Risk of bias in the individual studies was assessed independently by two reviewers using the Cochrane Handbook’s risk-of-bias tool (RoB2) for RCTs. ^51^ Any discrepancies or doubts were discussed and resolved together with a third reviewer. For each outcome in each study, all signaling questions were answered for the five bias domains: the randomization process, deviation from intended interventions, missing outcome data, measurement of the outcome, and selection of the reported result. For each domain the responses were collated to a final judgement. In addition, the overall judgement of either low risk of bias, some concerns or high risk of bias was assigned based on the five bias domains.

### Data synthesis and statistical analysis

To reduce the impact of bias due to missing data in the synthesis, we contacted the authors of the studies in case of missing estimates. In addition, in the data extraction we adhered to the best of our abilities to the intention-to-treat principle. ^52^ Meta-analyses were performed using Review Manager 5.4® (RevMan [Computer program]. Version 5.4. The Cochrane Collaboration, 2020). The effect measure of choice for the binary outcomes (e.g., postoperative complications) was the relative risk (RR) with 95% confidence intervals (95% CI) in order to assess the likelihood of the event happening in the experimental intervention group relative to the control comparator. ^53^ Secondary outcomes were expressed similarly except LoS and quality of life (continuous outcome measures), which were estimated based on the difference between means. ^54^ If data were presented as median and IQR, the assumption was that the mean approximately corresponded to the median while SD was estimated as IQR/1.35. ^55^

A meta-analysis for the relative risks of postoperative complications as well as for the secondary outcomes was conducted based on a random-effects model (modified Mantel-Haenszel) ^56^ and the results presented in Forest plots. The random-effects model was chosen beforehand, as we planned to include different types of surgical procedures and different types of interventions targeting two or more predefined risky SNAP factors; thus, this systematic review would not fulfil the criteria for using the fixed model for the meta-analysis. Clinical heterogeneity was considered and evaluated qualitatively, and the statistical heterogeneity was assessed as I ^2^-values as well as from the poor overlap in the visualized 95% CIs in the Forest plots. ^56^ We considered two-sided P-values less than 0.05 and 95% CIs for the RR values for binary outcomes that did not include 1 to be statistically significant.

According to the new guidelines by The Institute for Quality and Efficiency in Health Care (IQWiG) it is recommended to use a fixed-effect model in the case of very few studies, so we also conducted these for the purpose of sensitivity analysis on the primary outcome. ^57^

According to the original protocol, we also applied a network meta-analysis technique for postoperative complications in order to simultaneously compare three or more interventions in order to enable a comparison between multiple interventions since direct comparisons are limited. We estimated the odds ratios by default from the random effects network meta-analysis model with binomial likelihood and logit link. ^58^ For these (arm based) network meta-analyses, we used generalised linear mixed models combining a series of 2×2 tables, with the odds ratio modelled as a linear combination of study level covariates and random effects, representing variation between studies. ^59^

In addition, we planned subgroup analyses on different combinations of the five risky lifestyles, intensive interventions, elderly patient groups, and different types of surgery as well as sensitivity analyses.

Summary assessments of the quality of evidence for each important outcome were performed using the GRADE (Grading of Recommendations Assessment, Development and Evaluation) approach ^60^^,^^61^ which includes four levels of quality of evidence (high, moderate, low, and very low) and presented in a summary of findings table.

## Results

As illustrated in the PRISMA flowchart, the literature search revealed a total of 20,863 different records identified from various sources; these were screened for eligibility based on title and abstract, and 188 studies were subsequently assessed in full text. A total of 24 studies apparently met the inclusion criteria except for the criterion of at least two predefined risky SNAP factors. Consequently, we were only able to include two RCTs fulfilling our stringent eligibility criteria ^62^^,^^63^ ( Table 1).

**Table 1.** Full-text studies with multimodal interventions.

Author Year Country	Type of surgery (N ^o^ randomized)	SNAP inclusion criteria	SNAP intervention (intensity)	Additional intervention	Type of control	Postoperative complication definition	Complications IG vs. CG N _E_/N ^o^ included in analysis (%)
**Included: Two preidentified risk-factors **							
**Carli** ^62^ **2020 Canada**	Colorectal cancer resection (120)	N+P: *Frailty (Fried)*	P (I) N (B) S (B) A (B)	Psych. Int.	Rehab –programme identical to prehab.	Complications 30 days	See Figure 2
**Lauridsen** ^63^ **2022 Denmark**	Radical cystectomy for bladder cancer (13)	S (daily) + A (≥3u/day)	S (I) A (I)	-	Standard care	Complications 30 days	See Figure 2
**Excluded: One preidentified risk-factors **							
**Barberan-Garcia ** ^84^ **2018 Spain**	Major abdominal surgery Benign or malignant (144)	P: *Duke Activity Status Index*	P (I) N _m_ (B) S (B) A (B)	-	P (B) N _m_ (B) S (B) A (B)	Complications 30 days	19/62 (31) *vs.* 39/63 (62)
**Goodman** ^81^ **2007 UK**	Coronary artery bypass surgery (188)	N _o_: *BMI>28*	P (I) N (I)	Medication optimization	Standard care	Postoperative complications	Data not shown
**Kalarchian 2013** ^82^^,^^94^ **USA**	Bariatric surgery (240)	N _o_: *BMI>40 or BMI 35-40*	P (I) N _o_ (I)	-	Standard care: P (B) N (B)	Adverse events 30 days	1/71 *vs.* 0/72
**Liang** ^83^^,^^95^ **2018 USA**	Ventral hernia repair (118)	N _o_: *BMI 30-40 kg/m2*	P (I) N _o_ (I)	-	Standard counseling	30 days surgical site occurrences	3/44 (7) *vs* 6/34 (18)
**McIsaac** ^85^ **2022 Canada**	Intra-abdominal or thoracic cancer resection (204)	Frailty (Clinical Frailty scale)	P (B) N (B)	-	Standard care	In-hospital complications	42/94 (45) *vs.* 52/88 (59)
**Sawatzky** ^86^ **2014 Canada**	Coronary artery bypass graft (17)	P: *Sedentary*	P (I) N (I)	Education on risk factor management	Standard care	Operative + postoperative complications 30 days	4/8(50) *vs.* 4/7(57)
**Excluded: No preidentified risk-factors **							
**Allen** ^65^ **2022 UK**	Esophago-gastric cancer resection (54)	None	P (I) N (?)	Psych. and medical coaching	Standard care	Clavien–Dindo class ≥ IIIa	7/22 (32) *vs.* 12/23 (52)
**Ausania** ^66^ **2019 Spain**	Pancreatico-duodenectomy Malignant (48)	None	P (I) N _m_ (B)	-	Standard care	Complications 30 days	6/18 (33) *vs.* 12/22 (54)
**Bousquet-Dion ** ^67^ **2018 Canada**	Non-metastatic colon or rectal cancer resection (80)	None	P (I) N _m_ (B)	Anxiety-reduction	P (B) as rehab. N _m_ (B)	Complications 30 days	14/37 (38) *vs.* 8/26 (31)
**Ferreira** ^68^ **2021 Canada**	Lung cancer resection (124)	None	P (B) N (B) (prehab.+ rehab.)	Anxiety-reduction	P (B) N (B) (8 weeks rehab.)	Complications (Clavien-Dindo 1-5)	27/52 (52) *vs.* 26/43 (61)
**Fulop** ^69^ **2021 Hungary**	Colorectal resection Benign or Malignant (184)	None	P (I) N (B) A (B) S (B)	Anxiety-reduction	Standard care	Complications 30 days	17/77 (22) *vs.* 16/72 (22)
**Gillis** ^70^ **2014 Canada**	Colorectal cancer resection (89)	None	P (B) N _m+o_ (B) (prehab.+ rehab.)	Anxiety-reduction	P (B) N (B) (8 weeks rehab)	Complications 30 days	12/38 (32) *vs.* 17/39 (44)
**Kaibori** ^71^ **2013 Japan**	Liver resection Malignant (51)	None	P (?) N (B)	-	N (B)	Morbidity: Yes	2/25 (8) *vs.* 3/26 (12)
**Lawson** ^72^^,^^96^ **2021 Canada**	Thoracotomy for lung cancer (34)	None	P (I) N (B) S (B)	Anxiety-reduction	S (B)	Clavien–Dindo 1-3 ^+^ 30 days	11/20(55) *vs.* 4/8 (50)
**Liu** ^73^ **2020 China**	Thoracoscopic lobectomy Malignant (85)	None	P (B) N (B)	Mental relaxation skills	Standard care	Clavien–Dindo ≥ 2-3 ^+^ 30 days	4/37 (11) *vs.* 5/36 (14)
**López-Rodríguez-Arias ** ^74^ **2021 Spain**	Colorectal cancer resection (20)	None	P (B) N (B)	Anxiety-reduction	Standard care	Complications 30 days	2/10 (20) *vs.* 5/10 (50)
**Minnella** ^75^ **2018 Canada**	Esophago-gastric cancer resection (68)	None	P (B) N (B)	-	Standard care	Clavien-Dindo 1-5 complications 30 days	14/24 (58) *vs.* 18/25 (72)
**Minnella** ^76^ **2021 Canada**	Radical cystectomy Malignant (70)	None	P (B) N (B)	Anxiety-reduction	Standard care	Clavien-Dindo 1-5 complications 30 days	16/30 (53) *vs.* 16/28 (57)
**Molenaar** ^77^ **2023 Netherlands**	Colorectal cancer resection (269)	None	P (I) N (?) S(?)	Anxiety-reduction	Standard care	Complications 30 days	39/123 (32) *vs.* 54/128 (42)
**Nielsen** ^78^^,^^97^ **2008 Denmark**	Lumbar fusion (73)	None	P (B) S (I) A (I)	-	Standard care	Major complications 30 days	8/28 (29) *vs.* 8/32 (25)
**Nguyen** ^79^ **2022 France**	Total knee replacement (262)	None	P (I) N (B)	Anxiety-reduction	Standard care	Adverse events	?
**Stein** ^80^ **1970 USA**	Thorax, abdominal or other surgery (48)	None	P (I) - Chest physio. S (?)	Medication per indication	Standard care	Pulmonary complications grade 1-4	5/23 (44) *vs.* 15/25 (60)

**A:** Alcohol, **B:** Brief intervention, **BMI:** Body mass index, **CCI:** Comprehensive Complication Index, **CG:** Control group, **ERAS:** Enhanced recovery after surgery, **I:** Intensive intervention, **IG:** Intervention group, **Int:** Intervention **, N:** Nutrition, **N**_**E**_: Number of patients with event, **N** ^**o**^: Number of participants included in the study, **N**_**o**_: Obesity, **N**_**m**_: Malnutrition, **P:** Physical activity, **Physio:** Physiotherapy, **Prehab:** Prehabilitation (preoperative), **Psych:** Psychologic, **Rehab:** Rehabilitation (postoperative), **QoL:** Quality of life, **S:** Smoking, **u:** units **?**: No information available.

One of the two included studies was a multicentre RCT with stratification for smoking, alcohol and both had a small subgroup (n=10) investigating the effect of combined intensive smoking and alcohol cessation intervention for patients with these predefined risk factors undergoing radical cystectomy for bladder cancer. ^63^ In total, the sub-group consisted of 13 (eight in intervention group + five in control group) patients, but three did not undergo surgery leaving ten patients (seven in intervention group + three in control group) ( Table 2). The other included study (n=110) investigated the effect of a brief nutrition and intensive physical activity intervention on patients undergoing colorectal cancer surgery. ^62^ They included their patients based on being frail preoperatively according to the Fried Frailty Index, ^64^ which includes both elements of physical capability and malnutrition. Patients who were current smokers or had a risky alcohol intake were counselled regarding cessation, but no information regarding counselling content or lifestyle improvement were described ( Table 2). None of the two included studies received funding from foundations involved in any part of the trials (designing of study, study conduction, dissemination of results etc) except the funding.

**Table 2.** Study and baseline characteristics of the included studies.

Study	Carli 2020 ^62^		Lauridsen 2022 ^63^	
**Follow-up time**	Before surgery + 4weeks after surgery		6weeks + 3, 6, 12months after inclusion	
**Duration of intervention (protocol)**	4 weeks		6 weeks	
**Group allocation**	**IG**	**CG**	**IG**	**CG**
**Baseline to surgery days, median (IQR)**	40 (28-50)	35 (22-55)	8 (7-22)	13 (12.5-23)
**Adherence to intervention mean (SD)**	80% (27%) ^a^	-	100% ^b^	-
**Number of patients undergoing surgery**	55	55	7	3
**Men, N (%)**	29 (52.7)	23 (41.8)	7 (100%)	3 (100%)
**Age, median (IQR)**	78 (72-82)	82 (75-84)	63 (58-69)	71 (68-72)
**ASA score, N (%)**				
**II**	19 (34.5)	9 (16.4)	2 (28.5)	3 (100)
**III**	33 (60.0)	43 (78.2)	3 (43)	0
**IV**	3 (5.5)	3 (5.5)	0	0
**No information**			2 (28.5)	
**Current smokers, N (%)**	6 (10.9)	5 (9.1)	7 (100)	3 (100)
**Malnutrition, N (%)**	B: 27 (50.0) ^c^	B: 12 (26.7) ^c^	0 ^d^	0 ^d^
	C: 11 (20.4) ^c^	C: 13 (28.9) ^c^		
**BMI ≥ 30, N (%)**	14 (25.5)	16 (29.6)	1 (14.3)	0
**Risky alcohol use, N (%)**	5 (9.1) ^e^	3 (5.5) ^e^	7 (100) ^f^	3 (100) ^f^
**Physical active ≤ 30 min/day, N (%)**	-	-	2 (29)	1 (33)

**A:** Alcohol, **ASA:** American Society of Anesthesiologists, **au:** alcohol units, **BMI:** Body mass index, **CG:** Control group, **IG:** Intervention group, **IQR:** Inter quartile range, **min:** minutes, **N**: Number of, **SD:** Standard deviation.

^a^ Overall adherence to the programme (supervised and homebased; exercise and nutritional components).

^b^ Attendance rate at the 5 planned meetings.

^c^ Subjective global assessment. B indicates nutrition risk and C malnutrition.

^d^ Weight loss > 5% in the last month or food intake 0-25% of normal requirement in preceding week or BMI <18.5 + impaired general condition.

^e^ For men: (>15 standard drinks per week) and women: (>10 standard drinks per week).

^f^ ≥ 3 units of alcohol (36 g) daily.

### Risk of bias

**Figure 1.**
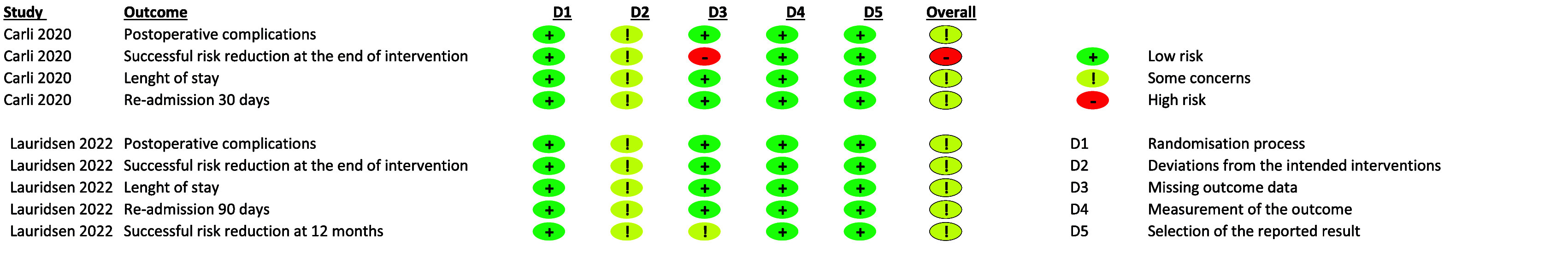
Risk of bias for included randomized controlled trials according to Cochrane’s risk of bias 2 tool. ^51^ +: low risk of bias, !: some concerns, -: high risk of bias, D1: randomization process, D2: deviations from the intended interventions, D3: missing outcome data, D4: measurement of the outcome, D5: selection of the reported result.

Risk of bias assessment for the two included studies are presented in Figure 1. Being behavioural intervention studies blinding of patients and study personnel was not possible. Otherwise, both studies were judged to have a low risk of bias in almost all domains for both primary and secondary outcomes. Exceptions were the secondary outcomes of successful risk reduction at the end of intervention in Carli et al. ^62^ and at 12 months in Lauridsen et al. ^63^ In Carli et al. less than 80% of the control group attended follow-ups. Furthermore, most of the non-attendances at follow-up were not justified. One could wonder whether the non-attendances at follow-up were the patients in the worst physical shape/with the least improvement, thereby inducing bias due to missing results. Therefore, the domain of missing outcome data was assessed as having a high risk of bias. The problems with follow-up data in Lauridsen et al. was explained by death of patients. One patient in each group died within 90 days due to complications, the rest due to recurrence of their bladder cancers, therefore the domain received some concerns instead of high risk of bias. To identify publication bias we planned to produce funnel plots but too few studies were identified for this.

### Postoperative complications

**Figure 2.**
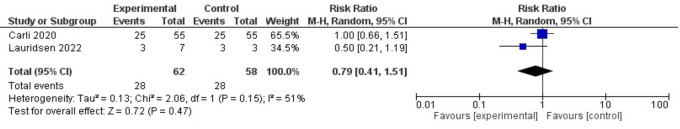
Forest plot with postoperative complications within 30 days. CI: confidence interval, df: degrees of freedom, P: p-value, Z: z-value.

None of the two included studies reported a statistically significant difference in postoperative complications within 30 days between intervention and control groups. Carli et al. ^62^ reported complications in 25 of 55 (45.5%) participants in both intervention and control group. Lauridsen et al. ^63^ reported complications in three of seven participants and three of three participants, respectively. The meta-analysis based on a random-effects model estimated a weighted relative risk of 0.79, 95% CI [0.41, 1.51] ( Figure 2). The meta-analysis based on a fixed-effect model estimated a weighed relative risk of 0.92, 95% CI [0.63, 1.34]; I ^2^: 51%.

According to the protocol, we performed a network meta-analysis comparing all intervention groups as if it had been compared in one large trial. When comparing an intensive (I) physical activity and brief (B) nutrition, smoking and alcohol cessation intervention (P _I_NSA _B_) with an intensive smoking and alcohol cessation intervention (SA _I_) the risk of complications could potentially be unfavorable for P _I_NSA _B_ (OR = 1.19 [0.26 to 5.45]). SA _I_ on the other hand might reduce the risk of complications when compared to treatment as usual (TAU) (OR = 0.79 [0.17 to 3.60]), but when TAU was compared to P _I_NSA _B_ there was no reason to suspect any difference in risk of complications (OR = 0.94 [0.46 to 1.92]).

### Secondary outcomes

**Figure 3.**
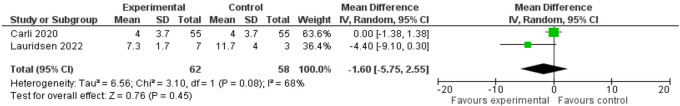
Forest plot with length of stay. CI: confidence interval, df: degrees of freedom, IV = inverse variance, P: p-value, SD: standard deviation, Z: z-value.

Carli et al. reported an identical length of stay in both groups of 4 days (mean difference 0.00, CI 95% [-1.38, 1.38]) while Lauridsen et al. reported length of stay of 7.3 days in the intervention group versus 11.7 days in the control group. The mean difference was -4.40 with CI 95% [-9.10, 0.30] pointing towards a shorter length of stay in the intervention group but still not statistically significant. Meta-analysis estimated a weighted mean difference of -1.60 days ( Figure 3).

It was not possible to conduct meta-analyses for the remaining secondary outcomes due to less than two studies reporting the outcomes. Carli et al. reported readmissions within 30 days in two of 55 (3.6%) participants versus five in 55 (9.1%) participants in the intervention and control groups, respectively (RR 0.40 [0.08, 1.97]). Lauridsen et al. reported readmissions within 90 days in five of seven participants versus two in two participants, respectively (RR 0.71 [0.45, 1.14]). Carli et al. did not report on long-term quality of life. Lauridsen et al reported one-year QoL and VAS QoL measured by the EQ-5D instrument. Mean difference for one-year QoL and VAS QoL was 0.06, 95% CI [-0.19, 0.32] and 2.4, 95% CI [-33.8, 38.6], respectively.

Regarding successful risk reduction, data were sparse and reported on few SNAP factors. All available results are reported as intention to treat in Table 3. Both studies reported no adverse events due to the interventions. The data available did not allow for any subgroup analyses to be made.

**Table 3.** Successful risk reduction of reported SNAP factors as intention to treat numbers.

Risk factor	Number of patients with successful risk reduction		RR [95% CI]	Study
	Intervention	Control		
*End of intervention*				
Smoking	3/7	1/3	1.29 [0.21, 7.89]	63
Alcohol	3/7	0/3	3.50 [0.23, 52.56]	63
Smoking and alcohol	3/7	0/3	3.50 [0.23, 52.56]	63
Physical activity (6MWT)	26/55	10/55	2.60 [1.39, 4.86]	62
*Long-term (1 year)*				
Smoking	1/7	0/3	1.50 [0.08, 29.15]	63
Alcohol	2/7	2/3	0.43 [0.10, 1.77]	63
Smoking and alcohol	1/7	0/3	1.50 [0.08, 29.15]	63

**CI:** confidence interval, **RR:** relative risk, **6MWT:** six-minute walk test.

### Certainty of evidence

The GRADE assessments of the quality of evidence for the meta-analysis outcomes for postoperative complications and length of stay are summarized in Table 4, including descriptions for downgrading the quality of the evidence.

**Table 4.** Summary of findings.

SNAP prehabilitation compared to treatment as usual in adult patients undergoing surgery						
Patient or population: Adult surgical patients Setting: Face-to-face interventions Intervention: SNAP prehabilitation Comparison: Treatment as usual						
Outcomes	Anticipated absolute effects ^*^ (95% CI)		Relative effect (95% CI)	N ^o^ of participants (studies)	Certainty of the evidence (GRADE)	Comments
	Risk with treatment as usual	Risk with SNAP prehabilitation				
Postoperative complications	483 per 1.000	**381 per 1.000** (198 to 729)	**RR 0.79** (0.41 to 1.51)	120 (2 RCTs)	⨁⨁◯◯ Low ^a^^,^^b^^,^^c^	
Length of Stay (LoS) follow-up: mean 30 days		MD **1.6 days lower** (5.75 lower to 2.55 higher)	-	120 (2 RCTs)	⨁◯◯◯ Very low ^c^^,^^d^	
Readmission 30 days	91 per 1.000	**36 per 1.000** (7 to 179)	**RR 0.40** (0.08 to 1.97)	110 (1 RCT)	-	
Readmissions 90 days	1.000 per 1.000	**710 per 1.000** (450 to 1.000)	**RR 0.71** (0.45 to 1.14)	9 (1 RCT)	-	

The individual successful SNAP-factor risk reductions were only reported in one study each.

* **The risk in the intervention group** (and its 95% confidence interval) is based on the assumed risk in the comparison group and the **relative effect** of the intervention (and its 95% CI).

**CI:** confidence interval; **GRADE:** The Grading of Recommendations Assessment, Development and Evaluation; **MD:** mean difference; **N ^o^:** Number of; **RCT:** Randomised controlled trial; **RR:** relative risk; **SNAP:** smoking, nutrition (overweight/obesity and malnutrition), alcohol, physical inactivity.

**GRADE Working Group grades of evidence.**

**High certainty:** we are very confident that the true effect lies close to that of the estimate of the effect.

**Moderate certainty:** we are moderately confident in the effect estimate: the true effect is likely to be close to the estimate of the effect, but there is a possibility that it is substantially different.

**Low certainty:** our confidence in the effect estimate is limited: the true effect may be substantially different from the estimate of the effect.

**Very low certainty:** we have very little confidence in the effect estimate: the true effect is likely to be substantially different from the estimate of effect.

**Explanations**.

^a^ Moderate statistical heterogeneity assessed as no serious inconsistency.

^b^ Downgraded for indirectness. Different SNAP-interventions being compared.

^c^ Downgraded for imprecision. Few patients and the confidence interval encompass both benefit and harm.

^d^ Downgraded for inconsistency. Substantial statistical heterogeneity.

## Discussion

Surprisingly, this systematic review identified only two randomized trials of prehabilitation before surgery targeting two or more co-existing and predefined risky SNAP factors (like physical activity below four hours per week and daily smoking) compared to treatment as usual or no treatment. More than 20 randomized trials on multimodal SNAP factor prehabilitation has been published and all but two had only one or no predefined risky SNAP factor as inclusion criteria. The lack of preidentified risk factors may result in populations with mixed needs for prehabilitation, thus, offering prehabilitation to both patients with and without risky SNAP factors thereby with or without a need for prehabilitation. We found no significant effect on postoperative complications in the two included studies.

The large majority of RCTs evaluating combined interventions mainly involved physical training and nutritional support, but they did not deliver the prehabilitation based on specified risky SNAP factors presented in the inclusion criteria ( Table 2). This contrasts with other prehabilitation programmes, e.g., to quit smoking and alcohol prior to surgery, which would not be offered to persons without risky alcohol or tobacco use.

Most of the excluded studies recruited participants on the basis of diagnosis or type of surgery rather than identified individual needs for prehabilitation. ^65^^–^^80^ Another six studies on combined interventions included participants based on only one predefined risky SNAP factor; overweight ^81^^–^^83^ or limited physical activity, function, or sedentary lifestyle. ^84^^–^^86^ In the 22 excluded studies, there seems to be a discrepancy between the patients’ needs for prehabilitation and the programmes delivered. This may explain the inconsistency in impact on postoperative complications.

Frailty is a medical syndrome characterized by “diminished strength, endurance, and reduced physiologic function”, ^87^ which leaves the individual with decreased tolerance of stressors and vulnerable to adverse outcomes. ^88^ Numerous screening tools exist, however, we have only included randomized trials using tools that include two or more of the predefined risk factors of relevance for this systematic review e.g. the Fried Frailty Index, ^64^ which involves weight loss and reduced physical activity in addition to muscular weakness, slow walking, and exhaustion. ^42^ The Fried Frailty Index is used in the included study by Carli et al. ^62^ and this frailty diagnosis is closely related to the development of postoperative complications. ^42^ However, the evidence from RCTs of frailty intervention is sparse, ^89^^,^^90^ but studies are ongoing. ^91^^,^^92^

The included randomized trial on intervention for co-existing risky alcohol and tobacco use prior to major surgery is based on the major risk reducing effect on the separate preoperative intensive smoking or alcohol intervention. ^12^^,^^32^^,^^33^ It is only a minor subgroup from a larger study, but we have not been able to identify other studies of combined intervention for these risk factors. However, studies on non-surgical patients have shown successful quitting with the combined intervention, thereby indicating that this could be successful in relation to surgery. ^93^

A strength in this review is the substantial search of electronic databases using a highly sensitive search strategy developed in consultation with an information specialist. Another strength is that we only included studies where participants were selected and offered interventions based on five predefined risk factors associated with increased risk of postoperative complications, specifically according to the individual patient’s risk profile. One may consider the inclusion of any type of surgery as a weakness, however, several of the risky SNAP factors have been shown to increase complication rates with around 50% across different kinds of surgery. ^11^^,^^13^^,^^16^

The limited number and the small size of the included studies provided a weak foundation for estimating the effect of combined prehabilitation. Especially, the combined alcohol and smoking cessation intervention constitutes a very small sub-group with unequal allocation to intervention- and control group despite of stratification, probably due to recruitment from five centres. ^63^ The other included study evaluated frailty intervention based on Fried Frailty Index with five parameters in total. However, it is not clear to which degree the two SNAP factors weight loss and low physical activity were present in the included participants. ^62^ None of the two included studies intervened or reported outcomes on all the risky SNAP factors identified in baseline data. The biases above further impact the limitation of this review. In addition, both studies were conducted on patient groups with specific cancers in Western high-income countries (Canada and Denmark). Therefore, the results must be interpreted with caution, and the generalisation should be carefully considered.

Prehabilitation aiming at combined risky SNAP factors seems to have potential to abate postoperative complications, and probably long-term health. This untapped potential has not been defined yet, which may have impact for the individual patients and their families as they will continue suffering from potentially avoidable complications after surgery.

Complications are also resource consuming, ^7^ adding to the workload of the health professionals, the economic burden on the healthcare system and the society at large. Economic resources often are limited. As those with risky SNAP have an increased risk of complications compared to those without risky SNAP factors, ^11^^–^^19^ it seems plausible that prioritizing SNAP prehabilitation to those with risky SNAP factors would likely derive the greatest benefit in terms of reduced postoperative complications. However, as this review shows, the effect of combined prehabilitation in patients with risky SNAP factors is still very uncertain. In the future, when more evidence is available, prioritizing those with the greatest benefit could potentially be relevant to minimize the socioeconomic costs of prehabilitation.

The findings of this review show a lack of studies providing combined prehabilitation based on patient needs present in the study population. Therefore, conduction of high-quality large-scale studies on combined prehabilitation targeting co-existing and predefined risky SNAP factors are strongly requested. The combined prehabilitation should build upon the individual patient’s needs from preoperatively identified SNAP factors to establish new evidence regarding impact on surgical outcome at short term and health on longer term. Future research should consider explicitly including a demand of predefined risk factors in the research question and in the inclusion criteria, ensuring that interventions are targeted to co-existing risky SNAP factors.

When designing the studies, researchers need to take into account the varying levels of im-plementation of ERAS that may add to narrowing the gap of the effect of the interventions. The standard care and ERAS components implemented vary across institutions, surgical specialties, and procedures. ^4^ This makes it complex to estimate the actual intervention effect and compare effects properly across studies. TAU should align with the standard care pro-vided at the specific institution and for the relevant procedure to ensure that the TAU group does not receive inferior care compared to patients not participating in the studies. As a re-sult, the definitions of TAU groups will vary between studies, highlighting the need for a de-tailed description of TAU content including specifying adherence to ERAS guidelines and the intensity of potential SNAP interventions. Additionally, the content of the intervention group including the intensity of the interventions should be described with equal detail. The thorough description and transparency is essential for enabling meaningful future meta-analyses. Furthermore, due to the complexity alternative statistical methods such as Bayesian metrics could be relevant to consider as they can provide a more nuanced understanding of intervention effectiveness beyond traditional p-values. ^98^

## Conclusion

This review only identified two small trials that did not demonstrate statistically significant effects on postoperative complications after prehabilitation targeting co-existing and predefined risky SNAP factors. Although surprising, this important finding highlights the lack of randomised trials evaluating the effect of individualised, combined prehabilitation programmes delivered in accordance with individual patients’ preoperative risky SNAP factors. The meta-analysis did not demonstrate statistically significant effects on postoperative complications in this context. However, the study underscores the need for more prehabilitation studies with interventions targeting preidentified risky SNAP factors in order to assess the true effect on postoperative complications.

## Author contributions

The corresponding author attests that all listed authors meet the ICMJE authorship criteria and that no others meeting the criteria have been omitted.

### Reporting guidelines

We have used the repository Open Science Framework for the PRISMA checklist and flowchart. The name of the project is ‘Impact on postoperative complications of combined prehabilitation targeting co-existing smoking, malnutrition, obesity, alcohol drinking, and physical activity: a systematic review and meta-analysis of randomised trials’. https://doi.org/10.17605/OSF.IO/RKAVF

Data are available under the terms of the Creative Commons Zero “No rights reserved” data waiver (CC0 1.0 Public domain dedication).

## Ethics and consent statement

Ethics and consent not required.

## Software availability statement

We used Review Manager 5.4® (RevMan [Computer program]. Version 5.4. The Cochrane Collaboration, 2020). for the meta-analysis. Review Manager 5.4® is no longer available for download, but a web version can be used with a paid licence. However, an open access alternative is OpenMeta[Analyst].

We used Covidence for the screening of references with a paid institutional license. However, a limited open access alternative is the web tool Rayyan.

## Data Availability

**We have used the repository** Open Science Framework for extended data ( complete search strings) and reporting guidelines (PRISMA checklist and PRISMA flowchart). ^99^ The name of the project is: Impact on postoperative complications of combined prehabilitation targeting co-existing smoking, malnutrition, obesity, alcohol drinking, and physical inactivity: a systematic review and meta-analysis of randomised trials. https://doi.org/10.17605/OSF.IO/RKAVF. Data are available under the terms of the Creative Commons Zero “No rights reserved” data waiver (CC0 1.0 Public domain dedication).
