# Supplementary material for: Impact on postoperative complications of combined prehabilitation targeting co-existing smoking, malnutrition, obesity, alcohol drinking, and physical inactivity: a systematic review and meta-analysis of randomised trials
Source: F1000Res. 2025 Apr 8;13:694. Originally published 2024 Jun 26. [Version 2] doi: 10.12688/f1000research.150880.2 (PMC12198731; doi:10.12688/f1000research.150880.2)
Supplement: Peer review history

# Reviewer report: Reviewer response for version 2

**DOI:** 10.5256/f1000research.177050.r391169

**Published:** 2025-06-25

**Authors:** Jamal Alshorman

**Affiliations:** 1 The Second Affiliated Hospital of Hainan Medical University (Ringgold ID: 477165), Haikou, Hainan, China; 2 Hubei University of Science and Technology, Xianning, Hubei, China

- **Comments to the Authors: **Your systematic review and meta-analysis on combined prehabilitation targeting SNAP factors is well-structured and addresses an important gap in surgical care. The rigorous methodology, including PRISMA guidelines and GRADE assessment, adds credibility. However, the limited number of included studies (only two) highlights a critical research void. Future studies should prioritize larger RCTs with clear definitions of TAU and ERAS integration to strengthen evidence. The focus on patient-tailored interventions is commendable, but broader inclusion of diverse surgical populations is needed.
- This manuscript fills a crucial niche by examining combined prehabilitation for multiple modifiable risks. The authors’ thorough search strategy and transparent reporting meet high methodological standards. While the small sample size limits conclusions, it underscores the urgent need for targeted research. The work effectively highlights gaps in current evidence, making a compelling case for future studies to prioritize patient-specific interventions. Indexing will advance the field by encouraging more focused trials on this understudied topic.

Are the rationale for, and objectives of, the Systematic Review clearly stated?

Yes

Is the statistical analysis and its interpretation appropriate?

Partly

If this is a Living Systematic Review, is the ‘living’ method appropriate and is the search schedule clearly defined and justified? (‘Living Systematic Review’ or a variation of this term should be included in the title.)

Yes

Are sufficient details of the methods and analysis provided to allow replication by others?

Yes

Are the conclusions drawn adequately supported by the results presented in the review?

Yes

Reviewer Expertise:

Orthopedics, spine, rehabilitation

I confirm that I have read this submission and believe that I have an appropriate level of expertise to confirm that it is of an acceptable scientific standard.

# Reviewer report: Reviewer response for version 2

**DOI:** 10.5256/f1000research.177050.r376735

**Published:** 2025-05-14

**Authors:** Tim Neumann

**Affiliations:** 1 Department of Anaesthesiology and Intensive Care Medicine; Campus Benjamin Franklin, Charité - Universitätsmedizin Berlin, Berlin, Germany

**Impact** **on postoperative complications of combined prehabilitation targeting co-existing smoking, malnutrition, obesity, alcohol drinking, and physical inactivity: a systematic review and meta-analysis of randomised trials** by Line N. Lydom, Sofie Anne-Marie S. Jensen, Susanne V. Lauridsen, Mette Rasmussen, Robin Christensen, Ulla N. Joensen, Jacob Rosenberg and Hanne Tønnesen

 This systematic review by Lydom and colleagues reflects now consistently the comparison of combined prehabilitation targeting predefined co-existing risky SNAP factors versus usual preoperative routine.

 The systematic review and meta-analysis were conducted meticulously with the highest quality. Among other issues, the research question and the study design were coherent. Study methods such as design and settings were well described according to the practice standards and reporting guidelines such as the Cochrane Handbook for Systematic Reviews of Interventions, PRISMA and AMSTAR, and the study was preregistered on PROSPERO before retrieval of data. The evidence was searched and reviewed systematically and comprehensively in a structured way by using multiple data sources of published data only. Analyses were clearly described. The risk of bias as well as external validity in the studies was addressed and discussed, the RoB2 instrument was used. Full-text studies with multimodal interventions were clearly presented. Included and excluded studies were sufficiently described. Statements about ethics, software availability and data availability as well as conflict of interest of all authors were provided. Data synthesis and statistical analysis followed current standards and was well justified and was thoroughly and conscientiously conducted. Meta-analyses were also conducted, even though only 120 patients from 2 studies from different settings could be included. Cochrane’s review manager and IQWiG recommendations were applied and quality of evidence was graded with the GRADE approach. Issues of funding and conflict of interest in respect to the included studies as well as for the review process were also well addressed. The evidence was clearly described and acknowledged, and it was discussed how this review might contribute to the further development care in this field.

 One comment about the rationale as described for conducting this review: The knowledge gap was well defined, however there are still questions from a clinician’s point of view in respect to the rationale for conducting this review: in what real-world clinical scenario, a caretaker or a patient or a patient advocate, a clinician or any health care provider would ask this question about the effect of a combined prehabilitation strategy targeting predefined coexisting risky SNAP factors against usual preoperative routines in surgical patients? Do the risks and the chances influence one another, add up or multiply? One assumption what happens if two or more risks were simultaneously identified and addressed was mentioned and one example was provided (Park et al., 2021) suggesting that a combination of being obese and smoking potentiates the risk of complications after surgery. However, this study with this search strategy was not designed to sufficiently address these issues. The usual approach to create high level evidence addressing SNNAP risks in a clinical context such as surgery is that one addresses one identified risk factor after the other. This approach was very helpful so far, e.g., to demonstrate that intensive interventions were more effective compared to brief interventions. Combing risk reducing strategies adds complexity and heterogeneity. Heterogeneity in available resources and interests of the care providing facility as the heterogeneity of the preferences, needs, and resources of every single patient and many unforeseeable interactions might further blur the picture. Considering 5 possible SNNAP interventions, 5! = 120 different combinations were possible to be compared versus “TAU”. Having a high degree of heterogeneity of possible settings and resources able to address the different needs of patients in mind, I still have no clear picture of how to establish new evidence regarding impact on surgical outcome at short term and health on longer term in this respect. To broaden the perspective, additional qualitative research and target group and stakeholder involvement as well as gender/sex issues might be considered in future research.

 However, since SNAP interventions are considered to be administered in the context of the needs of each patient in the surgical settings but are mainly studied in isolation in RCTs in order to deliver a useful comparison of the treatments studied, it was very helpful that this question has been investigated and carried out in this comprehensive manner.

 Thank You.

 Minor additional commends:

 Table 4 It was interesting that

 “Length of Stay (LoS) follow up: mean 30 days”:  there was no downgrading for indirectness, when different SNAP-interventions were compared.

Are the rationale for, and objectives of, the Systematic Review clearly stated?

Partly

Is the statistical analysis and its interpretation appropriate?

I cannot comment. A qualified statistician is required.

If this is a Living Systematic Review, is the ‘living’ method appropriate and is the search schedule clearly defined and justified? (‘Living Systematic Review’ or a variation of this term should be included in the title.)

Yes

Are sufficient details of the methods and analysis provided to allow replication by others?

Yes

Are the conclusions drawn adequately supported by the results presented in the review?

Partly

Reviewer Expertise:

perioperative medicine, health promotion, substance use and addiction medicine

I confirm that I have read this submission and believe that I have an appropriate level of expertise to confirm that it is of an acceptable scientific standard.

# Reviewer report: Reviewer response for version 1

**DOI:** 10.5256/f1000research.165488.r314669

**Published:** 2024-09-24

**Authors:** Tim Neumann

**Affiliations:** 1 Department of Anaesthesiology and Intensive Care Medicine; Campus Benjamin Franklin, Charité - Universitätsmedizin Berlin, Berlin, Germany

Lydom et al. conducted a systematic review and meta-analysis to examine the effect of combined prehabilitation in surgical patients targeting co-existing smoking, malnutrition, obesity, alcohol drinking, and physical inactivity (SNAP) on postoperative complications ≤30 days. Complications after surgery are highly relevant for patients as well as for the health system. Any reduction of complications is important. Studies addressing single SNAP risks with risk reducing interventions to patients undergoing operations have shown some benefit.

 It is unclear how two (!) or more interventions combined work for patients having two or more risk factor in comparison to treatment as usual or no treatment. This is a clinically relevant question for patients with two or more SNAP risk factors. It was of interest to learn from this study, that with this search strategy only two small randomized trials of prehabilitation before surgery targeting two or more co-existing and predefined risky SNAP factors were identified. This research question has not been addressed so far. 

 Patients before surgery were frail in one study, and were small subgroup with risky substance use (alcohol and smoking) in the other study. The effect of combined SNAP interventions on postoperative complications was found to be very uncertain.

 The following comments may be used to enhance the clarity and usefulness of this publication for the readers of this journal.

**Abstract Background, Introduction, Methods: Aim**

 There is a difference between the aim as stated in Abstract’s Background, the aim as stated at the end of Introduction, and the aim as outlined by the search strategy.

 The “combined prehabilitation” is mentioned in the aim in the introduction chapter, not in the Abstract Background, (here: “…prehabilitation targeting predefined co-existing risky lifestyle factors with usual preoperative routines in surgical patients”), whereas in the eligibility criteria it is phrased “at least two of the five predefined risky SNAP factors” and “combined intervention initiated preoperatively”. It would be helpful, if the aim is stated identical at all places.

**Introduction: Devising the research question**

 The rationale for comparing clinical pathways with interventions for patients with two or more factor and excluding those with only one SNAP risk factor might be better explained. Every patient with a defined risk should receive the appropriate intervention. Prehabilitation measures should be offered from a clinician’s point of view according to the patient needs and regardless of the number SNAP risks, and if provided, as intensive as needed. This systematic review addresses the clinical situation in which one needs the evidence that an offer of a combined intervention (regardless of SNAP risk combination) for those surgical patients only with 2 or more risk factors works better than treatment as usual (TAU). In this systematic review, studies reporting complications of one single SNAP factor only with one tailored intervention only were not considered and excluded from analysis. It is unclear why and whether two different interventions should work differently, when offered simultaneously. This might be discussed.

**Method:**

 Data synthesis and statistical analysis data extraction and meta-analytical procedures were done extensively and excellent along the Cochrane Collaboration guidelines, although the number of studies (n = 2) and included patients N = 103) was small.

**Method/Results:**

 The method of Risk Ratio Mantel-Haenszel random-effects method Figure 2 should be explained in the method section:  The RR calculation in in the Table 2 Forest plot with postoperative complications within 30 days in the Lauridsen et al (2022) study should be explained, as (3/7)/(3/3)=0.42.

 In the figure legends of the tables all abbreviations should be explained.

**Results, Table 1:**

 On missing word “end” in Table 1: “Lauridsen 2022 Successful risk reduction at the end of intervention”

**Discussion: Future research strategies**

 It was called for conducting high-quality large-scale studies, especially RCTs on combined interventions targeting individual co-existing and predefined risky SNAP factors. Will the findings of this SR be helpful devising future research? How would one take such results into account when devising the research question? It was also stated that “the combined intervention programs should build upon the individual patient needs from preoperatively identified SNAP factors and establish new evidence regarding impact on surgical outcome at short term and health on longer term”.

 Before an ethical RCT a prior probability about roughly 50:50 is assumed that the intervention works better. How TAU should be defined? It would be of interest, how the authors would suggest a study design, especially the TAU control condition after this systematic review and in the light of the evidence about single interventions. In the times of ERAS pathways, the TAU is not the same anymore as it was when SNAP risks were followed by a general 50% increased complication rate. How would one integrate existing and developing ERAS concepts into high-quality large-scale studies? How would one deal with the narrowing effect of intervention gap (Lauridsen et al. 2022) when designing this study?  Considering the variety of ERAS recommendations with outcome relevance, including the recommendation to address the SNAP risks, one must consider the intensity of each intervention, the reach into target population, the possible efficacy, effectiveness and efficiency, the staff’s adoption, settings, systems and communities variables, implementation consistency, costs and adaptions made during delivery, and maintenance/sustainment of intervention effects in individuals and settings over time. Very complex.

 When calculating sample size for future studies, would it be helpful to consider the findings of this review?

**Discussion: Socio-economic expenses of prehabilitation: only to those in need?**

 It was stated in the conclusion that this study underscored the importance of involving patient needs in future research and evidence-based strategies to optimize surgical outcomes. It was also stated in the discussion, that “offering effective interventions only to those in need of risk reduction will lessen the socio-economic expenses of prehabilitation”. Is this a conclusion from the findings of the study? Is this meant as a general statement, and /or is it based on previous findings, such as the studies in the reference list “65–80” as mentioned on page 13: “Most of the excluded studies recruited participants on the basis of diagnosis or type of surgery rather than identified individual needs for prehabilitations ^65–80^”? However, one has to keep in mind that in these studies the concept was to increase the functional capacity together with an evidence-based design to reduce perioperative stress and maintain physiological function and accelerate recovery after surgery and reduce complications. Activating patients in special holistic programs may also have other potential benefits.

 This should be to the benefit of all patients, regardless of whether individual risks exist.  Clarification is recommended.

**Conclusion, last sentence:**

 The importance of involving patient needs in future research and evidence-based strategies to optimize surgical outcomes is generally recognized. It does not become entirely clear how this study specifically underscores this.

**Discussion:**

 The limited number and the small size of the included studies is not a weakness, but an important finding: Research has not addressed this research question yet.

**Minor-**

**Introduction:**

 The authors called for risk reduction through targeted combined prehabilitation. This sentence might exclude those with a single SNAP risk in the context of this paper, which is most likely not intended by the authors. Rewording should be considered.

**Method:**

 Concerning the outcome variable “overweight” or “obesity”: To become non-overweight, non-obese can take very long, even when weight loss was achieved,  the patient would be still scored to be at risk. Not being at risk due to is difficult to achieve in severely obese patients, even when an intervention is effective, as weight reduction might take more time.

Are the rationale for, and objectives of, the Systematic Review clearly stated?

Partly

Is the statistical analysis and its interpretation appropriate?

I cannot comment. A qualified statistician is required.

If this is a Living Systematic Review, is the ‘living’ method appropriate and is the search schedule clearly defined and justified? (‘Living Systematic Review’ or a variation of this term should be included in the title.)

Yes

Are sufficient details of the methods and analysis provided to allow replication by others?

Yes

Are the conclusions drawn adequately supported by the results presented in the review?

Partly

Reviewer Expertise:

perioperative medicine, health promotion, substance use and addiction medicine

I confirm that I have read this submission and believe that I have an appropriate level of expertise to confirm that it is of an acceptable scientific standard, however I have significant reservations, as outlined above.

# Response

**Published:** 2025-04-02

**Authors:** Line Lydom

**Affiliations:** The Parker institute, Not Applicable, Frederiksberg, Denmark

Dear Reviewer

 Thank you very much for the peer-review and the valuable comments. We have addressed them one by one below. Changes in and additions to the article manuscript have been written in italics and marked changes to the text are underscored in the response sections below.

 We have uploaded an up-to-date version of the article including the changes below and including track changes:

**1.**

**Abstract Background, Introduction, Methods: Aim**

 There is a difference between the aim as stated in Abstract’s Background, the aim as stated at the end of Introduction, and the aim as outlined by the search strategy.

 The “combined prehabilitation” is mentioned in the aim in the introduction chapter, not in the Abstract Background, (here: “…prehabilitation targeting predefined co-existing risky lifestyle factors with usual preoperative routines in surgical patients”), whereas in the eligibility criteria it is phrased “at least two of the five predefined risky SNAP factors” and “combined intervention initiated preoperatively”. It would be helpful, if the aim is stated identical at all places.

Response:

  Thank you for noticing the differences. We appreciate your attentiveness as it of course is much clearer if the aim is stated identically at all places. Therefore, we have changed the aim in the abstract and at the end of the introduction section so that they are identical and include the terms “combined prehabilitation”, “predefined co-existing risky SNAP factors”, and “usual preoperative routines”.

 The aim is now stated both places as:

*This study aimed to compare the effect on postoperative complications of combined prehabilitation targeting predefined co-existing risky SNAP factors with usual preoperative routines in surgical patients.*

 The hypothesis at the end of the introduction section has also been revised so it uses the same terms:

*We hypothesised the combined prehabilitation based on predefined co-existing risky SNAP factors would reduce the postoperative complications compared to usual preoperative routines.*

 We have also revised the eligibility criteria in the methods section, as recommended, so they contain the exact same terms for clarity:

*We included randomised controlled trials enrolling populations of adult patients (≥18 years) undergoing any surgical intervention and having predefined co-existing risky SNAP factors, defined as at least two of the five risky SNAP factors (daily smoking, alcohol intake > 2 drinks daily (= above 24 g ethanol) or 14 drinks weekly, malnutrition (defined as either weight loss of 10-15% within the last 6 months, BMI < 18.5, Subjective Global Assessment (SGA) Grade C, Nutritional Risk Screening (NRS) > 5, or preoperative serum albumin < 30 g/l), BMI > 25 and physical activity < 4 hours/week), or as described by the authors and receiving a combined prehabilitation initiated preoperatively and targeting those lifestyles.*

**2.**

**Introduction: Devising the research question**

 The rationale for comparing clinical pathways with interventions for patients with two or more factor and excluding those with only one SNAP risk factor might be better explained. Every patient with a defined risk should receive the appropriate intervention. Prehabilitation measures should be offered from a clinician’s point of view according to the patient needs and regardless of the number SNAP risks, and if provided, as intensive as needed. This systematic review addresses the clinical situation in which one needs the evidence that an offer of a combined intervention (regardless of SNAP risk combination) for those surgical patients only with 2 or more risk factors works better than treatment as usual (TAU). In this systematic review, studies reporting complications of one single SNAP factor only with one tailored intervention only were not considered and excluded from analysis. It is unclear why and whether two different interventions should work differently, when offered simultaneously. This might be discussed.

Response:

 Thank you for the comments and reflections. We agree that patients with an identified need should receive prehabilitation regardless of the number of SNAP factors they have and that it should be offered as intensive as needed. The effect of several of the single snap factor interventions have been investigated previously in systematic reviews or addressed in guidelines referencing existing studies (reference 13, 32, 33 in the review). Furthermore, many patients are now living with more than one SNAP factor which seems to potentiate the risk of complications after surgery. For example, a study by Park et al. found that the combination of being obese and smoking at the same time potentiated the risk of complications after surgery. Therefore, we have chosen that the focus of this review would be to investigate the effect of combined prehabilitation targeting preidentified co-existing risky SNAP factors. Also, we expected many studies investigating combined prehabilitation interventions to have an eligibility criterion of the SNAP factors being intervened on to be preidentified in their population. Therefore, we were very surprised to find only two studies. In retrospect, it would have been interesting to include studies on single SNAP interventions to see if these studies targeted predefined SNAP factors and the effect of the interventions or if they were conducted on mixed populations SNAP-wise. Unfortunately, this is not within the scope of this review and not possible to add due to the search string but it would be very interesting to investigate in a future systematic or scoping review.

 To make the rationale and our chosen scope of this review clearer we have added the following elaboration of the potentiated effect of two risky SNAP factors in the introduction just before the aim section:

*This is despite of up to half of hospital patients have co-existing SNAP factors, ^39 – 41^ such as smoking and overweight or frailty involving both malnutrition and physical inactivity which significantly potentiate the risk at surgery. ^14 , 42^ For example Park et al. found that the combination of being obese and smoking at the same time potentiated the risk of complications after surgery. ^14^ As the risk of the two risk factors in combination seems to potentiate each other, it points even more towards the relevance of investigating the effect of combined prehabilitation.*

**3.**

**Method:**

 Data synthesis and statistical analysis data extraction and meta-analytical procedures were done extensively and excellent along the Cochrane Collaboration guidelines, although the number of studies (n = 2) and included patients N = 103) was small.

**Method/Results:**

 The method of Risk Ratio Mantel-Haenszel random-effects method Figure 2 should be explained in the method section:  The RR calculation in in the Table 2 Forest plot with postoperative com-plications within 30 days in the Lauridsen et al (2022) study should be explained, as (3/7)/(3/3)=0.42.

Response:

 The reviewer raises a valid point regarding the need for a clearer explanation of the risk ratio (RR) calculation in the Mantel-Haenszel random-effects method and its application in Figure 2.

 We understand that the manually calculated RR for the smaller study is approximately 0.43, based on the crude event rates (3/7 vs. 3/3). However, the RR reported in our meta-analysis (0.5) was derived using the Mantel-Haenszel method with random effects, as implemented in RevMan. This incorporates a correction that results in slight differences between manually calculated crude RRs and those derived via meta-analytic methods, particularly in small or sparse datasets, where the RR is reduced. Overall, the larger dataset, the closer to the crude rate (and the smaller confidence interval). E.g., the Lauridsen Study: RR = 0.50 [0.21, 1.19], a hypothetic 10 times larger study with similar results: 0.44 [0.33, 0.57] or 100 times larger: 0.44 [0.33, 0.57]. Therefore, the discrepancy between the manual RR (0.43) and the RevMan-reported RR (0.5) arises due to differences in calculation approach.

 We have now updated the data synthesis and statistical analysis subsection in the methods section so it includes the description of the reason for and the use of the modified Mantel-Haenszel random-effects method for meta-analysis:

*A meta-analysis for the relative risks of postoperative complications as well as for the secondary outcomes was conducted based on a random-effects model (modified Mantel-Haenszel)* *^56^* *and the results presented in Forest plots.* *The random-effects model was chosen beforehand, as we planned to include different types of surgical procedures and different types of interventions targeting two or more predefined risky SNAP factors; thus, this systematic review would not fulfil the criteria for using the fixed model for the meta-analysis.*

 Furthermore, we have rearranged the order of the text sections in this subsection.

**4.**

 In the figure legends of the tables all abbreviations should be explained.

Response:

 Thank you for the comment. We can see that in the online version the table legends have disappeared. They are present when the article is downloaded as a pdf format. When we downloaded the article as a DOC-file, the table legends with explanations of the abbreviations disappeared again. We can see how it is confusing.

 We have reviewed all legends for the figures and tables and made sure all abbreviations are explained in the legends. In the new updated article we have added all explanations of the abbreviations again.

**5.**

**Results, Table 1:**

 On missing word “end” in Table 1: “Lauridsen 2022 Successful risk reduction at the end of intervention”

Response:

 Thank you very much for noticing the missing word. We believe it is in Figure 1 were we can see that you are right that it is missing. We have added “end” to “Lauridsen 2022 Successful risk reduction at the *end* of intervention” in Figure 1.

**6.**

**Discussion: Future research strategies**

 It was called for conducting high-quality large-scale studies, especially RCTs on combined interventions targeting individual co-existing and predefined risky SNAP factors. Will the findings of this SR be helpful devising future research? How would one take such results into account when devising the research question? It was also stated that “the combined intervention programs should build upon the individual patient needs from preoperatively identified SNAP factors and establish new evidence regarding impact on surgical outcome at short term and health on longer term”.

Response:

 Thank you for the valuable reflections and these questions. We believe that the findings of this review highlight the need for considering including predefined risk factors in the research question and the inclusion criteria in future research and we have added this reflection at the end of the discussion section (including small revisions of the text already there):

*The findings of this review show a lack of studies providing combined prehabilitation based on patient needs present in the study population. Therefore* *,* *conduction of high-quality large-scale studies on combined prehabilitation targeting co-existing and predefined risky SNAP factors are strongly requested. The combined prehabilitation should build upon the individual patient’s needs from preoperatively identified SNAP factors to establish new evidence regarding impact on surgical outcome at short term and health on longer term. Future research should consider explicitly including a demand of predefined risk factors in the research question and in the inclusion criteria, ensuring that interventions are targeted to co-existing risky SNAP factors.*

**7.**

 Before an ethical RCT a prior probability about roughly 50:50 is assumed that the intervention works better. How TAU should be defined? It would be of interest, how the authors would suggest a study design, especially the TAU control condition after this systematic review and in the light of the evidence about single interventions. In the times of ERAS pathways, the TAU is not the same anymore as it was when SNAP risks were followed by a general 50% increased complication rate. How would one integrate existing and developing ERAS concepts into high-quality large-scale studies?

Response:

 We agree that the ethical perspective is important. While there is some evidence for the superiority of prehabilitation targeting some of the single risky SNAP factors, uncertainty still exist whether combined prehabilitation targeting co-existing SNAP factors will provide superior benefits. A study using standard care as it is structured at the specific institution ensures that no harm is done to the TAU group by withholding care compared to not participating in the study. The TAU group should, therefore, be allowed to receive potential SNAP interventions if it is part of the standard care at the institution so that they do not receive worse care than if not participating in the study.

 We have added the following reflections on how the TAU group should be defined at the end of the discussion section:

*When designing the studies, researchers need to take into account the varying levels of implementation of ERAS that may add to narrowing the gap of the effect of the interventions. The standard care and ERAS components implemented vary across institutions, surgical specialties, and procedures. ^4^ This makes it complex to estimate the actual intervention effect and compare effects properly across studies. TAU should align with the standard care provided at the specific institution and for the relevant procedure to ensure that the TAU group does not receive inferior care compared to patients not participating in the studies. As a result, the definitions of TAU groups will vary between studies, highlighting the need for a detailed description of TAU content including specifying adherence to ERAS guidelines and the intensity of potential SNAP interventions. Additionally, the content of the intervention group including the intensity of the interventions should be described with equal detail. The thorough description and transparency is essential for enabling meaningful future meta-analyses.*

**8.**

 How would one deal with the narrowing effect of intervention gap (Lauridsen et al. 2022) when designing this study?  Considering the variety of ERAS recommendations with outcome relevance, including the recommendation to address the SNAP risks, one must consider the intensity of each intervention, the reach into target population, the possible efficacy, effectiveness and efficiency, the staff’s adoption, settings, systems and communities variables, implementation consistency, costs and adaptions made during delivery, and maintenance/sustainment of intervention effects in individuals and settings over time. Very complex.

Response:

 We agree that it is very complex, and that the intervention gap is narrowing. There are increasing things to consider and include when designing a future study including all the mentioned above. This is why it is imperative to report the abovementioned aspects in detail in study reports for transparency and so other researchers can evaluate the use of the results properly. We have tried to address this in the additions mentioned in the response to comment number 7. Furthermore, we have added the following in continuation of the additions described in response 7:

*Furthermore, due to the complexity alternative statistical methods such as Bayesian metrics could be relevant to consider as they can provide a more nuanced understanding of intervention effectiveness beyond traditional p-values (new reference: Seretny 2022).*

**9.**

 When calculating sample size for future studies, would it be helpful to consider the findings of this review?

Response:

 Thank you for the relevant comments asking how to use the findings of this systematic review. Since we only found two small RCTs to include and the effect of the meta-analysis  was deemed very uncertain, this review does not offer an adequate foundation for calculating sample size for future multimodal prehabilitation studies. Until further evidence becomes available, sample size calculations should still be guided by estimated effects derived from single-modality studies investigating prehabilitation targeting preidentified risk factors and be approached with caution and conservatism.

**10.**

**Discussion: Socio-economic expenses of prehabilitation: only to those in need?**

 It was stated in the conclusion that this study underscored the importance of involving patient needs in future research and evidence-based strategies to optimize surgical outcomes. It was also stated in the discussion, that “offering effective interventions only to those in need of risk reduction will lessen the socio-economic expenses of prehabilitation”. Is this a conclusion from the findings of the study? Is this meant as a general statement, and /or is it based on previous findings, such as the studies in the reference list “65–80” as mentioned on page 13: “Most of the excluded studies recruited participants on the basis of diagnosis or type of surgery rather than identified individual needs for prehabilitations65–80”? However, one has to keep in mind that in these studies the concept was to increase the functional capacity together with an evidence-based design to reduce perioperative stress and maintain physiological function and accelerate recovery after surgery and reduce complications. Activating patients in special holistic programs may also have other potential benefits.

 This should be to the benefit of all patients, regardless of whether individual risks exist.  Clarification is recommended.

Response:

 We agree that holistic programs may offer other potential benefits beyond reducing complications. Patients without risky SNAP factors may still benefit from training or nutritional interventions to, probably, a lesser degree or in areas other than reducing the risk of complications. However, economic resources are often limited, making it important to identify which patients would benefit the most from prehabilitation and to understand the effects of SNAP interventions specifically in these patients, rather than in a mixed population of patients with and without high-risk SNAP factors. The phrase “offering effective interventions only to those in need of risk reduction will lessen the socio-economic expenses of prehabilitation” is intended as a general statement about the possibility of prioritizing resources for those who would derive the greatest benefit from the intervention. It is not directly a finding from our study. We have tried to clarify this by making the following revisions in the discussion section:

*Complications are also resource consuming, ^7^ adding to the workload of the health professionals, the economic burden on the healthcare system, and the society at large. Economic resources often are limited. As those with risky SNAP have an increased risk of complications compared to those without risky SNAP factors, ^11 – 19^ it seems plausible that prioritizing SNAP prehabilitation to those with risky SNAP factors would likely derive the greatest benefit in terms of reduced postoperative complications. However, as this review shows, the effect of combined prehabilitation in patients with risky SNAP factors is still very uncertain. In the future, when more evidence is available, prioritizing those with the greatest benefit could potentially be relevant to minimize the socioeconomic costs of prehabilitation.*

**11.**

**Conclusion, last sentence:**

 The importance of involving patient needs in future research and evidence-based strategies to optimize surgical outcomes is generally recognized. It does not become entirely clear how this study specifically underscores this.

Response:

 Thank you for pointing this out. We agree that maybe the phrasing is a bit wrong. The study does not directly underscore the importance but rather show a lack of studies where prehabilitation is offered based on present risk factors in the population. This is needed to assess the effect of the interventions on patients who have the given risky SNAP factors. To make this clearer, we have revised the end of the conclusion:

*The* meta-analysis did not demonstrate statistically significant effects on postoperative complications in this context. *However, the study underscores the need for more prehabilitation studies with interventions targeting preidentified risky SNAP factors in order to assess the true effect on postoperative complications.*

**12.**

**Discussion:**

 The limited number and the small size of the included studies is not a weakness, but an important finding: Research has not addressed this research question yet.

Response:

 Thank you for the comment. We agree that the limited number and small population size is not a weakness of the study in general, but more a weakness related to being able to estimate the effect of combined prehabilitation in this review.

 We have changed the phrasing to the following:

*The limited number and the small size of the included studies provided a weak foundation for estimating the effect of combined prehabilitation.*

 Furthermore, we have made an addition to the conclusion to highlight the surprising finding of a lack of studies evaluating combined prehabilitation targeting predefined risky lifestyles:

*This review only identified two small trials that did not demonstrate statistically significant effects on postoperative complications after prehabilitation targeting co-existing and predefined risky SNAP factors. Although surprising, this important finding highlights the lack of randomised trials evaluating the effect of individualised, combined prehabilitation programmes delivered in accordance with individual patients’ preoperative risky SNAP factors. The meta-analysis did not demonstrate statistically significant effects on postoperative complications in this context. However, the study underscores the need for more prehabilitation studies with interventions targeting preidentified risky SNAP factors in order to assess the true effect on postoperative complications.*

**13.**

**Minor-**

**Introduction:**

 The authors called for risk reduction through targeted combined prehabilitation. This sentence might exclude those with a single SNAP risk in the context of this paper, which is most likely not intended by the authors. Rewording should be considered.

Response:

 Thank you for this remark. In a clinical context, we agree that prehabilitation should include those patients living with a single risky SNAP factor. We believe that prehabilitation should target all risky SNAP factors the patient has, not just one if they have more. Therefore, we believed the effect of combined prehabilitation to be worth investigating in this paper and chose to exclude studies only intervening on 1 SNAP factor. We have rephrased the sentence as part of the changes made in regard to comment 2.

**14.**

**Method:**

 Concerning the outcome variable “overweight” or “obesity”: To become non-overweight, non-obese can take very long, even when weight loss was achieved, the patient would be still scored to be at risk. Not being at risk due to is difficult to achieve in severely obese patients, even when an intervention is effective, as weight reduction might take more time.

Response:

 We agree and fully acknowledge that achieving a non-overweight or non-obese status can take considerable time and may not be feasible for all patients with severe obesity during the prehabilitation period. Consequently, this outcome is likely to reflect the effects of the interventions primarily on individuals not too far from the thresholds of being overweight or obese depending on the length of prehabilitation. As a result, the observed effects may substantially underestimate the true impact of the weight-loss interventions. To better capture the benefits of these interventions for individuals who are unlikely to completely abandon their unhealthy lifestyles, we also included a secondary outcome focused on any positive change in lifestyle. However, for future studies, it would probably be beneficial to revise and refine this outcome to assess improvements more effectively in risky lifestyle behaviors.

 If some of the included studies had included a weight-loss intervention, the design of the outcome could have been identified as a limitation in the discussion section. However, since neither of the two included studies examined weight-loss interventions, we do not believe this point would add significant value to the paper. Therefore, we have not addressed it in the manuscript.
